# COVID-19 and the Gut Microbiome: More than a Gut Feeling

**DOI:** 10.1128/mSystems.00453-20

**Published:** 2020-07-21

**Authors:** Daniel van der Lelie, Safiyh Taghavi

**Affiliations:** a Gusto Global LLC, Morrisville, North Carolina, USA; Princeton University

**Keywords:** COVID-19, gut microbiome

## Abstract

Due to its fundamental role in the induction, training, and function of the immune system, it is critical to include characterizations of the gut microbiome in clinical trials and studies that aim to broaden our understanding of coronavirus disease 2019 (COVID-19). Understanding the “gut-lung axes,” where gut microbiome composition influences the lung’s susceptibility to viral infections and viral infections of the lung alter gut microbiome composition toward proinflammatory functional dysbiosis, will be critical in addressing COVID-19, including disease progression, the importance of preexisting conditions, and the risk for developing complications. These insights may further help to develop better intervention strategies for COVID-19 and other diseases caused by respiratory viruses.

## Abstract (precis)

Due to its fundamental role in the induction, training, and function of the immune system, it is critical to include characterizations of the gut microbiome in clinical trials and studies that aim to broaden our understanding of coronavirus disease 2019 (COVID-19). Understanding the “gut-lung axes,” where gut microbiome composition influences the lung’s susceptibility to viral infections and viral infections of the lung alter gut microbiome composition toward proinflammatory functional dysbiosis, will be critical in addressing COVID-19, including disease progression, the importance of preexisting conditions, and the risk for developing complications.

## PERSPECTIVE

Over the last 2 decades, three coronaviruses have emerged as causes for serious respiratory tract infections in humans: severe acute respiratory syndrome (SARS) caused by SARS coronavirus 1 (SARS-CoV-1), which emerged in late 2002 and disappeared by 2004; Middle East respiratory syndrome (MERS) caused by MERS-CoV, which emerged in 2012 and remains in circulation in camels; and coronavirus disease 2019 (COVID-19) caused by SARS-CoV-2, which emerged in December 2019 and has had considerable global economic and health impacts. Approximately 20% of patients with COVID-19 develop serious complications, especially an overactive immune response resembling cytokine storm syndrome (CSS) (1), often accompanied by fatal acute respiratory distress syndrome. Other serious or fatal complications of COVID-19 include hypercoagulation, and in young children a rare condition, multisystem inflammatory syndrome in children (MIS-C), that resembles Kawasaki disease (2).

## 

### Treatment and intervention strategies.

Current treatment and intervention strategies being developed for COVID-19 focus on vaccine development for disease prevention, and in the absence of an approved vaccine, on treatment strategies that address viral replication or decrease the severity of complications caused by an overactive immune system. These strategies include remdesivir (Gilead Sciences), a nucleoside analogue that inhibits SARS-CoV-2 RNA-dependent RNA polymerase activity and viral replication (3), immune inhibitors, like the KEVZARA trial in which the efficacy of an anti-interleukin 6 (IL-6) receptor antibody is evaluated to stop an overactive inflammatory response, and development of monoclonal antibodies as therapy, as well as ongoing efforts with immediately available convalescent plasma (4). Also, type 1 interferons (especially alpha interferon [IFN-α] and IFN-β) with broad antiviral activity are being evaluated in clinical trials to treat SARS-CoV-2 (5, 6). *In vitro* studies successfully confirmed the sensitivity of SARS-CoV-2 to type 1 interferons (7).

### Respiratory viruses and the gut microbiome.

The role of the gut microbiota in the severity of viral respiratory tract infections, such as those caused by the influenza virus (8), was recently recognized. Lung stroma were identified as the target of microbiota-driven signals (9) that set the type 1 interferon (IFN-I) signature in these cells as a defense against early viral infection. Influenza viruses replicate almost exclusively in the respiratory tract, yet infected individuals may also develop gastrointestinal (GI) symptoms as observed for COVID-19 patients (10). These symptoms resulted from significantly changed gut microbiome composition through a mechanism dependent on type 1 IFN-I molecules. Notably, influenza-induced IFN-I molecules produced in the lung promoted depletion of obligate anaerobic bacteria and enrichment of *Enterobacteriaceae* in the gut, leading to a proinflammatory dysbiotic gut environment (11), further influencing virus infectivity. Changes to a proinflammatory gut microbiome have also been observed for other respiratory viruses, including adenovirus. Several commensal taxa essential for a healthy gut microbiome decreased, whereas genera containing potential pathogens, such as *Neisseria*, increased in abundance upon adenovirus infection in a lemur model (12). It should be noted that gut microbiome dysbiosis is often an underlying condition in patients suffering from diabetes, obesity, and autoimmune and aging-related diseases, the highest risk groups for severe COVID-19 (13), and that GI symptoms are often indicative of severe COVID-19 complications.

A dysbiotic gut environment and epithelial inflammation increases levels of angiotensin-converting enzyme 2 (ACE2), a cell surface receptor that plays a key role in dietary amino acid homeostasis, innate immunity, and gut microbial ecology (14). ACE2 is the target of SARS-CoV-2 (15), and elevated ACE2 as found in patients characterized by a (preexisting) proinflammatory gut microbiome creates conditions favorable for infection by coronaviruses (16) like SARS-CoV-2 of the gut epithelium, from which it can further spread through the body (17). This is consistent with the development of GI infections and detection of viral RNA in the feces of many COVID-19 patients, including those who tested negative by PCR of their respiratory secretions (18).

A unique subgroup of COVID-19 patients has been described as characterized by mild disease severity and marked by the presence of digestive symptoms. These patients were found to be more likely to test positive for viral RNA in stool, to have a longer delay before viral clearance, and to experience delayed diagnosis compared with patients with only respiratory symptoms (19). Prolonged digestive symptoms, especially diarrhea, are negatively associated with gut microbiota richness and composition (20), alterations that are closely associated with immune dysfunction, which might explain the delayed viral clearance. This implicates that reshaping the gut microbiota may be an adjuvant therapy in patients suffering from digestive symptoms linked to COVID-19.

A recent study from Wuhan, China, confirmed a link between gut microbiome composition and the predisposition of healthy individuals to COVID-19 (21). Increased levels of *Lactobacillus* species correlated to higher levels of anti-inflammatory IL-10 and improved disease prognosis; increased levels of proinflammatory bacterial species, including *Klebsiella*, *Streptococcus*, and Ruminococcus gnavus correlated with elevated levels of proinflammatory cytokines and increased disease severity. These species were previously found as enriched in the proinflammatory gut environment of patients suffering from a range of conditions, including diabetes, obesity, irritable bowel disease (IBD), and high blood pressure. Kawasaki disease, a condition similar to MIS-C that is increasingly reported as a complication in young children diagnosed with COVID-19 (2), is characterized by a dysbiotic gut microbiome with increased levels of *Streptococcus* and decreased levels of *Lactobacillus* species compared to healthy children (22). Therefore, COVID-19-induced changes in gut microbiome composition might contribute to this complication.

### Discussion.

A clear link may exist between gut microbiome health and COVID-19 progression. Understanding the “gut-lung axes,” where gut microbiome composition influences the lung’s susceptibility to viral infections and viral infections of the lung alter gut microbiome composition toward proinflammatory functional dysbiosis will be critical in addressing COVID-19, including disease progression, the importance of preexisting conditions, and the risk for developing complications. These insights will help to identify biomarkers and targets for drug development, and to develop intervention strategies based on live biotherapeutics and nutrition to overcome gut microbiome dysbiosis and restore intestinal homeostasis as contributing factors to COVID-19.
